# Elemental diets role in treatment of high ileostomy output and other gastrointestinal disorders 

**Published:** 2015

**Authors:** Kamran Rostami, David Al Dulaimi

**Affiliations:** *Gastroenterology Unit, Alexander Hospital Redditch, UK*

**Keywords:** Elemental diets, Ileostomy GI disorders, Ulcerative colitis, Stoma

## Abstract

Elemental diet (ED) has been used widely in the treatment of gastrointestinal disorders, especially with the management of Crohn's disease. This modality of diets provides all essential nutrients, and contains protein in the form of free amino acids that are theoretically easily absorbed. High output ileostomies are a rare but important complications of stoma formation following bowel surgery. Treatments could be challenging and include anti-diarrhoeals, octreotide and proton pump inhibitors. There is very little research regarding the use of elemental diets in the treatment of patients with post-operative high ileostomy outputs. Adequate management of high output ileostomies might prevent significant morbidity. In this case report, we describe a patient who underwent a subtotal colectomy for ulcerative colitis complicated by refractory high ileostomy output despite maximal standard medical therapy for years. The ileostomy output was dramatically reduced following the introduction of an elemental diet. This case suggests a possible role for the introduction of an elemental diet in the management of high output ileostomies. Besides presenting this case with high output ileostomy, we reviewed the role of ED in other gastrointestinal disorders.

## Introduction

 Elemental diet Extra (EDE) is proposed as a standard therapy in the treatment of Crohn’s disease (CD) (1-4). But their role in managing patients with refractory high output ileostomies is not studied yet. EDE consists of a diet that contains all the patient’s nutritional requirements, including protein, fats, sugars and vitamins. The protein requirement is provided as free essential and non-essential free amino acids. Elemental diets have been shown to be effective in inducing and maintaining remission in inflammatory bowel disease (IBD), especially CD (5). The beneficial effect of an ED for patients with CD is widely accepted, but the mechanism underlying the efficacy remains uncertain. Proposed mechanisms include an alteration of bacterial flora, possibly due to the low antigenicity of amino acids compared to proteins, the low fat content and improvement of nutritional status are all postulated as possible explanations of efficacy of ED in CD (6). 

**Table 1:** Elemental diet indication in gastrointestinal disorders

Studies used in this paper	Study outcome	No patients	Reference
UC/CD using ED enriched with Fish oil (Ulcerative colitis and Crohn)	IL-1ra increased	7UC, 5CD	38
Chronic pouchitis (ulcerative colitis)	Improving diarrhoea 12x=>6x/day	12	14
Crohn’s disease	Inducing remission and Improving nutritional state	Multiple studies	1-2, 5, 32, 36
Ulcerative colitis	Inducing remission	23	34
Chronic pancreatitis	Pain and nutrition indices	596	12
Recurrent aspiration pneumonia in patients with PEG	Reduced number of pneumonia	127	11
Eosinophilic oesophagitis	Mucosal improvement in 4 weeks	18	15
High ileostomy output	Reduced output and concentration of sodium, bile acids and trypsin	10	26
Refractory coeliac disease	Histological improvement in 8/8	10	30
Atopic eczema	Improvement in the eczema in 27/37 (73%)	37	27

Considering alterations in bacterial flora, it seems reasonable to suggest that dietary measures leading to an alteration of the intestinal flora could result in a decrease in the production of toxins that induce inflammation compared to a normal diet, leading to clinical improvement. This hypothesis is supported by animal studies suggest that an ED leads to a reduction in the amount and diversity of intestinal microbiota and decrease of pro-inflammatory cytokines via a change in composition of lactic acid producing bacteria (7-10). In this article we present a patient with a HOI refractory to the maximal standard treatment that responded to the introduction of an ED and a review of current literature assessing the role of ED in other gastrointestinal disorders. 

## Case Report

A 70 years old lady with ulcerative colitis (UC) was treated with a subtotal colectomy in ten years ago and was subsequently seen in the medical gastroenterology clinic with a HOI, refractory to conventional treatment including codein phosphate, loperamide, PPI and octerotide. In addition to her UC with HOI, she had significant co-morbidities including psoriatic arthritis and type 2 diabetes and osteoporosis. She had been emptying her ileostomy bag 8 times during the day and 5 during the night since she underwent a subtotal colectomy. Furthermore she had troublesome excoriations, erosions and bleeding around the ileostomy fistulae. Her life quality was severely impaired with significant psycho-social difficulties. Investigations including ileoscopy were entirely normal including histology aside from blood tests showing a chronically raised urea and white cell count. She was treated with an ED (the 028 diet). Following introduction of the ED her symptoms improved in less than 48 hours. After initiating the exclusive ED her ileostomy bag requires changing 3 times per day and does not need to be changed overnight. The stomal erosions, excoriations and bleeding all improved and healed in space of few days. In keeping with an improvement in ileostomy function, her quality of life and psycho-social wellbeing improved (table 2).

**Table 2:** The benefit of ED in this case

Reduced Ileostomy output
No need to empty the ileostomy bag over night
Stop leaking and eliminate the need of showering over night
Ensure a good quality of sleep, leading to a better energy level and mood improvement
Healing the excoriation, erosions and stopping the bleeding around fistulae
Long term improving of absorption of nutrient

## Discussion

We suggest that this patient’s improvement in symptoms and reduction in ileostomy output was due to the introduction of an ED diet. To further support this suggestion we re-introduce a normal diet and continue with her previous treatment regimen the symptoms have returned. The elemental diet was the only intervention that reduced her Ileostomy output during 12 years’ time. We postulate that the ED diet led to a dramatic reduction in ileostomy output by reducing inflammation and facilitating nutrient absorption, thereby reducing osmotic load. The current literature suggest ED might be useful in patients requiring gastrostomy, those with underlying pancreatic and oesophageal diseases. An ED promotes rapid gastric empting and this may explain the mechanism underlying the reported reduction in gastrostomy associated aspiration pneumonia, compared to standard liquid diets (11).

 A study has reported an improvement in quality of life for patients with chronic pancreatitis treated with an oral low-fat elemental diet composed of purified amino acids (12). An ED have also been used successfully in chylous ascites (13) occurring following an anterior resection and and chronic pouchitis (14). A further study has reported an improvement in patients with eosinophilic oesophagitis, treated with an ED. Interestingly the improvement in symptoms was associated with an objective histologic improvement (15).

Complications following ileostomy include infection, thrombo-embolism, stomal obstruction, stomal prolapse, local erythema and inflammation. A high output ileostomy (HOI) is a rare complication that occurs post-operatively when there is persistent, excessive and disproportionate fluid and electrolyte loss through the stoma. In the first few days after ileostomy formation, there is frequently an increased stool effluent through the stoma, but this rapidly decreases because of ‘ileostomy adaptation (16). A HOI occurs when stoma output remains high. HOI can result in severe dehydration and electrolyte imbalance (17,18). Traditional treatments include anti-diarrhoeal agents, proton pump inhibitors and octroetide. The role of elemental diet in management of patients with HOI remains uncertain and ED might have a role in improving the life quality of at least some of these patients. Traditional treatments include anti-diarrhoeal agents, proton pump inhibitors and octroetide. The role of elemental diet in management of patients with high ileostomy output remains uncertain. This case highlight a possible role for this modality of management of this condition. A large randomised study will required to assess accurately the role of ED in patients with refractory HOI. 

ED has been reported to be beneficial for patients with a variety of gastrointestinal disorders as described below.

**Peri-operatively**

Many patients require special nutritional support following surgery, and elemental diets may assist in the management of such patients (19). The property of minimal residues of elemental diets may also be of value in colonic and rectal surgery where a reduction in the faecal output and local inflammation may assist healing (20-23).

**Fistulas and skin**

The use of residue-free elemental diets for human nutrition in the treatment of catabolic states was reported by Stevens and Randall (1969) (24, 25) and the application to the management of chronic gastrointestinal fistulae has so far been one of the most successful uses of this form of therapy. Ileostomy output of fluid and electrolyte is less and the corrosive nature of the discharge is also reduced (26). Elemental diet therapeutic effect has been proven in dermatology conditions as well. A strict antigen avoidance regimen has been reported to be associated with improvement of atopic eczema where conventional treatments have failed (27).

**Malabsorption states**

The nature of elemental diets, requiring little or no digestion in the upper alimentary tract and no micelle formation, offers a potential advantage in the management of patients with maldigestion and malabsorption states. The use of an elemental diet following an initial period of intravenous feeding would appear to be the treatment of choice in such patients at present (28, 29). Elemental diet is also known as a therapeutic option that can provide long-term immunopathologic and clinical improvement in refractory coeliac disease (30, 31). Olaussen et al reported histologic improvement and reduced epithelial IL-15 in a series of 8 patients. They noted clinical improvement 6/8 patients, with 1 patient showing normalization of hypoalbuminemia. Three patients were able to stop total parenteral nutrition (32).

**Elemental diet in treatment of Crohn’s disease**

ED’s were initially used for CD in the early 1970s as a form of nutritional support before intestinal surgery (1, 5, 21). The treatment not only improved the nutritional state of patients but also induced remission from disease activity (2, 33, 34).

Subsequently, EDs have been shown to be as successful as corticosteroids (2, 35) and total parental nutrition (36) in reducing the clinical activity of the disease. ED appear to influence the balance between pro- and anti-inflammatory cytokines; increasing anti-inflammatory to pro-inflammatory cytokine ratio in favour of a reduction in inflammation. This anti-inflammatory effect may not be specifically due to the amino acid composition of an ED, as diets containing casein have similar anti-inflammatory effects (6). 

**Elemental diet in treatment of ulcerative colitis**

The evidence for the effectiveness of ED in UC is not as strong as in CD. Few studies have reported on the use of an ED in the management of UC. It is possible, that in severe exacerbations of the disease elemental diet therapy with its quality of low residue would be of some value. Axelsson et al. reported in a cohort of 23 patients with UC and 11 patients with CD patients who had steroid refractory disease. In this group 44% of both UC and CD patients went into remission after introducing elemental diet. From this group 6 of 13 patients with UC treated with ED remained well 7-28 months after the study (34). The published trials investigating the effect of an ED in UC are involving small patient numbers (37). Unfortunately, for unsubstantiated reasons, nutritional therapy such as an ED, may remain underutilized, even in paediatric patients, who are most vulnerable to the harmful effects of nutrient deficiencies on growth, pubertal development, and bone health.

**Chronic pancreatitis**

An oral low-fat elemental diet may improve chronic pancreatitis patients' quality of life.

Kataoka at al reported ingesting a low-fat elemental diet composed of purified amino acids for 12 weeks reduced the pain remarkably in 596 patients with chronic pancreatitis. They also found significant improvements in nutritional indices in these patients (12).

Recommendations for using ED’s in gastrointestinal disorders are impaired by a lack of good quality randomised trials, with most evidence arising from case series and small poor quality studies. An ED may be an effective, yet often underused, treatment for numerous gastrointestinal disorders. An ED contain nutrients in simple forms (such as amino acids, simple carbohydrates, fats, vitamins and minerals) and requires little or no digestion to take place prior to absorption. As an ED does not contain potentially antigenic proteins, it may be that pro-inflammatory antigens are avoided (38). An ED can also improve nutritional status, further supporting enhanced mucosal healing in inflammatory disorders. In addition to a reduction in inflammation an ED may reverse the increased gut permeability associated with mucosal inflammation and the short bowel syndrome. Furthermore the stoma site may benefit as the stoma output may be altered and less likely to cause local inflammation (26). 

The published trials investigating the effect of an ED diet for patients with UC suggest a positive treatment effect. The current BSG guidelines (39) recommend against using ED in short bowel syndrome with high ileostomy output. This recommendation was based on a study consisted of 7 patients only (40). Prospective controlled studies with large number of patients are required to evaluate the efficacy of an ED for patients with HOI and UC.

## Note

Please see the pages: 4-5
